# Tumor-Associated Macrophages in Tumor Immunity

**DOI:** 10.3389/fimmu.2020.583084

**Published:** 2020-12-03

**Authors:** Yueyun Pan, Yinda Yu, Xiaojian Wang, Ting Zhang

**Affiliations:** ^1^Department of Radiation Oncology, Second Affiliated Hospital, School of Medicine, Zhejiang University, Hangzhou, Zhejiang, China; ^2^Institute of Immunology, School of Medicine, Zhejiang University, Hangzhou, Zhejiang, China

**Keywords:** tumor-associated macrophages, regulation, immunosuppression, tumor microenvironment, tumor therapy

## Abstract

Tumor-associated macrophages (TAMs) represent one of the main tumor-infiltrating immune cell types and are generally categorized into either of two functionally contrasting subtypes, namely classical activated M1 macrophages and alternatively activated M2 macrophages. The former typically exerts anti-tumor functions, including directly mediate cytotoxicity and antibody-dependent cell-mediated cytotoxicity (ADCC) to kill tumor cells; the latter can promote the occurrence and metastasis of tumor cells, inhibit T cell-mediated anti-tumor immune response, promote tumor angiogenesis, and lead to tumor progression. Both M1 and M2 macrophages have high degree of plasticity and thus can be converted into each other upon tumor microenvironment changes or therapeutic interventions. As the relationship between TAMs and malignant tumors becoming clearer, TAMs have become a promising target for developing new cancer treatment. In this review, we summarize the origin and types of TAMs, TAMs interaction with tumors and tumor microenvironment, and up-to-date treatment strategies targeting TAMs.

## Introduction

Macrophages play critical roles in both innate and adaptive immunity and are known for their remarkable phenotypic heterogeneity and functional diversity. Embryonic hematopoietic stem cells in a variety of tissues during fetal development and differentiate into tissue-specific resident macrophages, including Kupffer cells in the liver, alveolar macrophages in the lung, and osteoclasts in bone tissue. After birth, bone marrow-derived precursors in particular circulating monocytes can also differentiate into macrophages in steady state or during tissue inflammation (1). Macrophages are involved in tissue and systemic inflammation and immunity, as well as tissue reconstruction. They have a wide range of functions, including phagocytosis, antigen presentation, defense against microbial cytotoxicity, and secretion of cytokines, complement components, etc. (2). It is worth noting that the broad biological activities of macrophages often have diametrically opposite characteristics, such as inflammatory response and anti-inflammatory activity; immunogenic and inducing immune tolerance; causing tissue destruction and repairing (3).

Tumor-associated macrophages (TAMs) are macrophages that participate in the formation of the tumor microenvironment. TAMs are widely present in various tumors (4). TAMs can promote tumor growth, invasion, metastasis, and drug resistance (5). It has been proposed that functional difference of macrophages is closely related to the plasticity of macrophages, and its functional phenotype is regulated by molecules in tumor microenvironments.

In this review, we discuss the origins and types of TAMs, the interaction between tumors and the tumor microenvironment, and review the emerging strategies for cancer treatment *via* targeting TAMs.

## Origins and Types of TAMs

### Origins

For a long period of time, it is believed that macrophages in tumors are exclusively recruited from the periphery by chemotaxis and generated by monocytic precursors in the local environment. However, more recent evidence shows that at least certain tumors, tissue-specific embryonic-derived resident macrophages infiltrate tumor tissues and thus represent a nonnegligible input source of TAMs (6). Although there have been studies showing that monocytic-derived but not embryonic-derived resident macrophages are capable in supporting the growing body of TAMs in the inflammatory environment of tumor, the potentially different roles of monocytic- versus embryonic-derived TAMs on tumor development and/or progress remains an intriguing question that is largely unanswered (2).

M-MDSCs (monocyte-related myeloid-derived suppressor cells) are currently known as another main circulating precursor of TAMs. MDSCs are a type of myeloid leukocytes that is related to immunosuppression (7). Based on surface markers Ly6C+/Ly6C- and Ly6C-/Ly6G+, MDSCs can be divided into monocyte (M)-related and granulocyte (G)-related MDSC. Among them, M-MDSCs are induced into TAMs by various chemokines (8).

It is all know that macrophages derive from bone marrow-derived monocytes. In tumors, TAMs mainly originate from bone marrow monocytes, but recent evidence suggests that, recruitment of circulating monocytes is essential for TAMs accumulation. Circulating inflammatory monocytes could be recruited by multiple chemokines (CCL2 and CCL5) and cytokines (CSF-1 and members of the VEGF family) to tumor (9). Tumor growth can also induce the differentiation of CCR2+ monocytes into TAMs (10).

Furthermore, complement components, particularly C5a, are an important mediator of the recruitment and functional polarization of TAMs (11). Indeed, such chemokines do more than attractants do because they activate transcription programs that help macrophages tilt toward the functional of a particular phenotype (12). At the same time, CSF-1 is a monocyte attractant, as well as macrophage survival and polarization signals, which drive TAM to immunosuppressive differentiation M2 macrophages (13). Unlike CSF-1, GM-CSF activates macrophage function associated with antitumor activity (14).

### Types

Macrophages undergo specific differentiation in different tissue environments, and can be divided into two different polarization states: M1 type macrophages (M1) and M2 type macrophages (M2).

M1 can respond to dangerous signals transmitted by bacterial products or IFN-γ, which attracting and activating cells of the adaptive immune system; an important feature of M1 is that it can express nitric oxide synthase (iNOS) and reactive oxygen species (ROS) (15–17) and cytokine IL-12 (18). M1 also has the function of engulfing and killing target cells.

M2 expresses a large number of scavenger receptors, which is related to the high-intensity expression of IL-10, IL-1β, VEGF and matrix metalloprotein (MMP) (19, 20). M2 has the function of removing debris, promoting angiogenesis, tissue reconstruction and injury repairments, as well as promoting tumorigenesis and development (4).

It is worth noting that the polarization of macrophages into M2 appears to be oversimplified. Some people have classified M2 macrophages into M2a (induced by IL-4 or IL-13), M2b (induced by immune complexes combined with IL-1β or LPS) and M2c (induced by IL-10, TGFβ, or glucocorticoid), and M2d (conventional M2 macrophages that exert immunosuppression) (21, 22).

## The Role of TAMs in Tumor Progress

**Figure 1:**
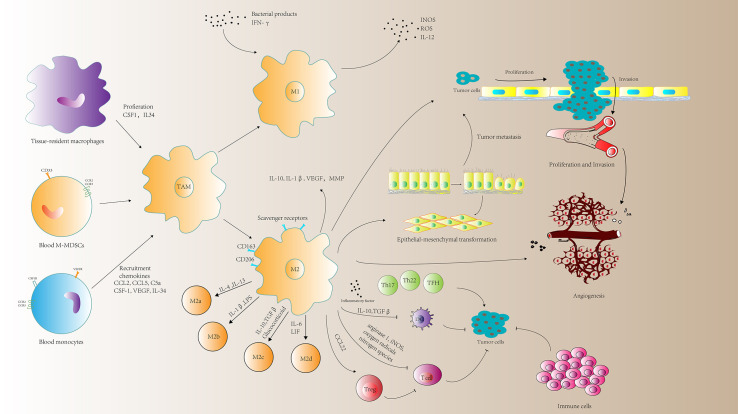
A schematic representation of the roles of tumor-associated macrophages (TAMs) in tumor progression. TAMs can mediate immune response, tumor cell proliferation and invasion, angiogenesis and metastasis. MMP, matrix metalloprotein; M-MDSCs, monocyte-related myeloid-derived suppressor cells; CSF1, macrophage colony-stimulating factor; VEGF, vascular endothelial growth factor; ROS, reactive oxygen species; INOS, nitric oxide synthases; LIF, leukocytosis induced factor.

Current studies have shown that TAM population is in a state of constant transition between the two forms of M1 and M2 type. The proportion of each form is determined by the type and concentration of different signals in the tumor environment (**Figure 1**).

### M1 Macrophages and Tumor Suppression

M1-type macrophages have anti-tumor effects, which can distinguish tumor cells from normal cells. By identifying tumor cells and ultimately killing tumor cells, studies have found that M1 type macrophages have two different effects on killing tumor cells mechanism. M1 type macrophages directly mediate cytotoxicity to kill tumor cells: macrophage-mediated cytotoxicity is a slow process (generally requires 1 to 3 days) and involves multiple mechanisms. For example, macrophages release tumor killing molecules such as ROS and NO, which have cytotoxic effects on tumor cells (23). The other is antibody-dependent cell-mediated cytotoxicity (ADCC) killing tumor cells: ADCC requires less time to kill tumor cells (generally within a few hours) and requires the participation of anti-tumor antibodies (24).

### M2 Macrophages Promote Tumor Cell Proliferation and Invasion

TAM infiltration is closely related to tumor cell proliferation. Many studies have shown that TAMs can express a variety of cytokines that stimulate tumor cell proliferation and survival, including epithelial growth factor (EGF), platelet-derived growth factor (PDGF), TGF-β1, hepatocyte growth factor (HGF), and epithelial growth ligands of the factor receptor (EGFR) family and basic fibroblast growth factor (BFGF) (25). The ligands of the EGFR family play an important role in tumorigenesis, especially breast and lung cancers. Members of this family can form homo- or heterodimers on the cell surface, mediating the transduction of cell proliferation signals. In all, TAMs are an important cell source for EGF secretion in tumor tissues (25).

As for invasion, in glioma cells, extracellular adenosine deaminase protein cat eye syndrome critical region protein 1 (CECR1) has been shown to regulate the maturation of macrophages. CECR1 is induced by M2-like TAM secretory effects activate MAPK signaling and stimulate the proliferation and migration of glioma cells (26). Another investigation shows that a positive feedback loop of CCL5 and CCL18 between TAMs and myofibroblast is constituted to drive the malignant invasion of phyllodes tumor (PT). CCL5 binds to CCR5, and activates the AKT signal to recruit and repolarize TAMs. TAMs release CCL18 to further induce the invasion of malignant PTs by differentiating the mesenchymal fibroblasts to myofibroblast, causing the malignancy of PTs (27).

### TAMs Promote Tumor Metastasis

Tumor metastasis is an important feature of poor prognosis after tumor therapy. The main reason for tumor cell migration and metastasis is the degradation and damage of tumor tissue endothelial cell basement membrane. It has been reported that activated TAMs exert a direct effect on promoting metastasis *via* directly producing soluble factors (28). M2 macrophages can destroy matrix membrane of endothelial cells by secreting matrix metalloproteinases (MMPs), serine proteases, cathepsins, and decompose various collagen and other components of extracellular matrix, thereby helping the migration of tumor cells and tumor stromal cells (19, 20). Epithelial-mesenchymal transition (EMT) is the basis of tumor metastasis (29). This process enables tumor cells to acquire the ability to migrate and endows them with the properties of stem cells (30). Besides, cytokines produced by tumor cells also promote the differentiation process of TAMs, thus forming a positive feedback loop between TAMs and EMT (31).

### M2 Macrophages Promoting Angiogenesis

TAMs are enriched in hypoxic areas with poor blood supply (1). Proangiogenic effects by TAMs involves the coordinated regulation of a wide range of cytokines, including BFGF, VEGF, IL-1, IL-8, TNF-α, MMP-9, MMP-2, and nitric oxide (NO). The coordinated expression of these molecules promotes the proliferation of endothelial cells, matrix remodeling and vascularization in time and space. Macrophages can release the angiogenic molecules and express a series of enzymes involved in the regulation of angiogenesis, including MMP-2, MMP-7, MMP-9, MMP-12, and cyclooxygenase-2 (20, 32).

However, metabolism still exists in angiogenesis, and it is still unknown whether changes in metabolism affect these functions. Hypoxic TAM strongly up-regulates the expression of mTOR’s negative regulator REDD1. REDD1-mediated mTOR inhibition can hinder glycolysis in TAM and reduce its excessive angiogenic response, thereby forming abnormal blood vessels (33).

### Immune Regulation by TAMs

TAM can regulate the killing effect of T cells and NK cells on tumor cells. M1 macrophages increased the number of total and activated natural killer (NK) cells in fibrotic liver, released TNF-related apoptosis-inducing ligand (TRAIL), and induced HSC apoptosis (34). HCC-derived exosomes induced macrophages to upregulate the expression of IFN-γ and TNF-α in T cells, while the expression of inhibitory receptors PD-1 and CTLA-4 was upregulated (35). In mesothelioma, the macrophages isolated from pleural effusion showed the M2 phenotype were negatively correlated with T cells *in vivo*, which emphasized the use of macrophages as treatments in mesothelioma Target potential (36).

In addition to these functions, TAMs can also directly inhibit CD8^+^ T-cell proliferation through metabolism of L-arginine *via* arginase 1, iNOS, oxygen radicals or nitrogen species (37–39). Besides, TAMs recruit Tregs through CCL22 (40), which further suppress the antitumor immune response of T-cells. Conditional TAM ablation blocks Treg cell recruitment and inhibits tumor growth by lowering the CCL20 level of xenograft mice (41).

Substantial evidence indicates that the inflammatory reaction at a tumor site can promote tumor growth and progression. Inflammation and immune evasion are considered as hallmarks of cancer. It has been reported that TAMs can also contribute to cancer-related inflammation that leads to tumorigenesis by generation of inflammatory Th subset such as TFH (42). Toll-like receptor 4 (TLR4)-induced monocyte inflammation is important for induction of IL21+ TFH-like cells, which operate in IL21-IFNγ-dependent pathways to induce plasma cell differentiation and thereby create ideal conditions for M2b macrophage and cancer progression (42) (**Figure 1**). These suggest that strategies to influence functional activities of inflammatory cells may benefit anticancer therapy.

## Factors Regulating TAMs Functions

**Figure 2:**
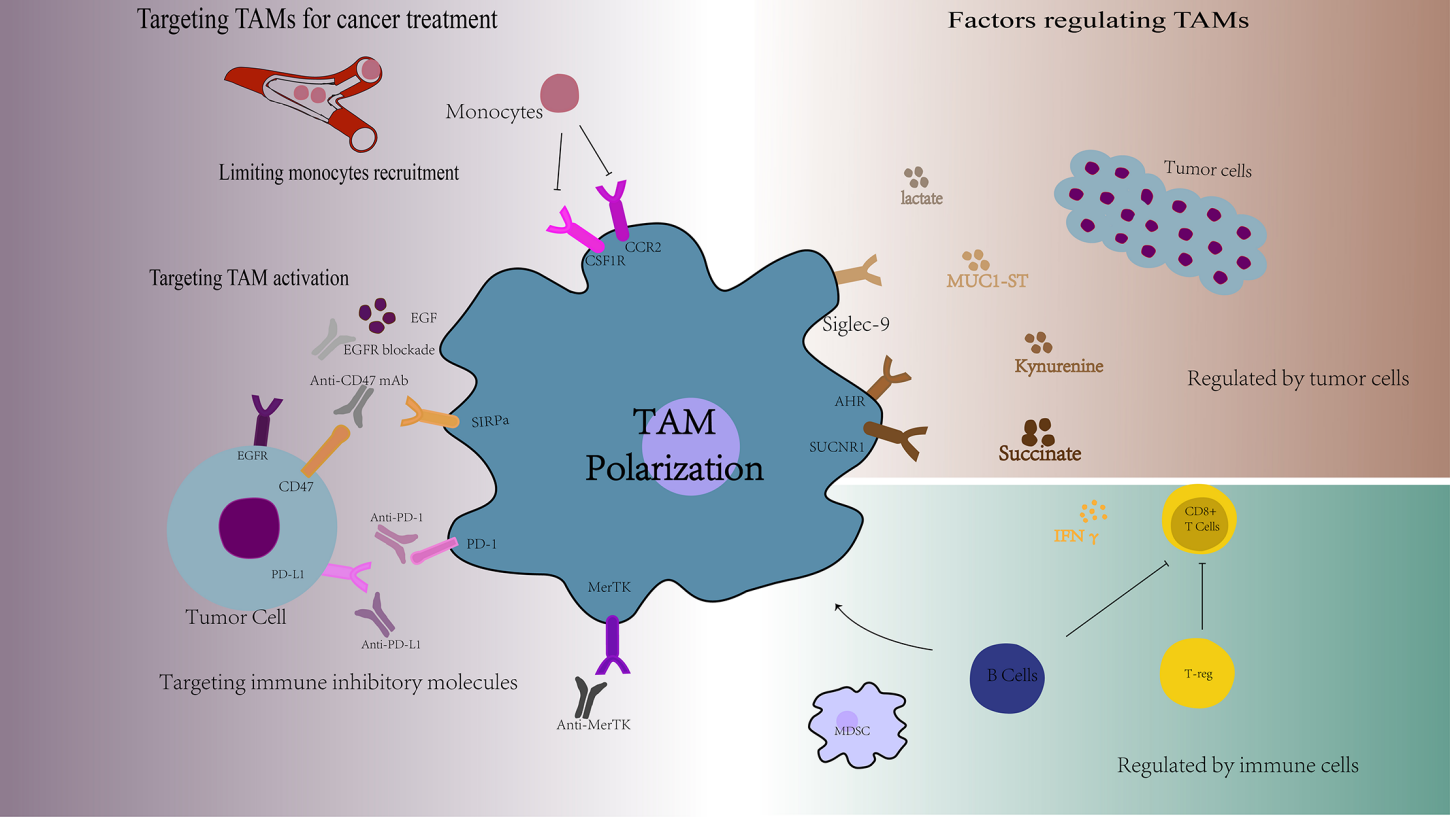
Overview of the factors regulating TAMs functions and the targets of TAMs for cancer treatment. TAMs are a collection of multiple cell types with a wide range of functional effects, which are regulated by many different factors, such as the tumor cell-derived soluble molecules, tumor metabolic alterations, and other immune cells. Targeting TAMs is a new cancer treatment strategy, including limiting monocytes recruitment, targeting TAMs activation, and targeting TAMs specific markers. AHR, aromatic hydrocarbon receptor; SUCNR1, succinate Receptor 1; EGF, epidermal cell growth factor; SIRPα, signal regulatory protein alpha.

TAMs are a collection of multiple cell types with a wide range of functional effects under steady state and pathological conditions. This diversity is regulated by many different factors, such as the tumor cell-derived soluble molecules, tumor metabolic alterations, other immune cells and other factors (**Figure 2**).

### Tumor Cell-Derived Soluble Molecules

TAMs can be activated and polarized by tumor cell-derived soluble molecules, thereby promoting tumor progression and metastasis. Tumor cells secrete the sonic hedgehog (SHH), and tumor-derived SHH drives TAM M2 polarization. Hh-dependent polarization of TAM suppresses the recruitment of CD8^+^ T cells to TME *via* inhibiting CXCL9 and CXCL10, mediating TAM immunosuppression mechanism (43). In addition, kynurenine produced by glioblastoma cells can activate the aromatic hydrocarbon receptor (AHR) in TAMs, and AHR can drive KLF4 expression and inhibit NF-κB activation in TAMs, which regulate TAM function and T cell immunity (44). Cancer cells can also release succinate into their microenvironment and activate the succinate receptor (SUCNR1) signal, thereby polarizing macrophages to TAMs (45). Meanwhile, there is a positive correlation between the expression of osteopontin (OPN) in tumor cells and TAMs infiltration. OPN promotes chemotaxis migration and activation of TAMs (46). Also, when mucin MUC1 is expressed on cancer cells and is decorated with multiple short, sialylated O-linked glycans (MUC1-ST), which will induce TAM to express M2-like phenotype (47).

### Tumor Metabolic Alterations

It is worth noting that macrophage polarization is correlated with distinct metabolic characteristics pertaining to glucose metabolism (48, 49), lipid metabolism (50), and glutamine metabolism (51). Such metabolic alterations can also determine the phenotype and function of TAMs in promoting the cancer progression (52).

Cancer cells can utilize metabolic byproducts to take the control of tumor-infiltrating immune cells to their own benefit. For example, lactate secreted by glycolysis in cancer cells, which transfers the polarization of TAMs from a pro-inflammatory (M1-like) to an anti-inflammatory (M2-like) phenotype (53, 54). Another research shows that membrane cholesterol efflux drives TAM reprogramming and tumor progression. Ovarian cancer cells promote membrane cholesterol efflux, and increased cholesterol efflux promotes IL-4 mediated signaling in TAMs, which will promote tumor invasion and metastasis (55). In addition, glutamate-ammonia ligase (GLUL) favors M2-like TAMs polarization by catalyzing the conversion of glutamate into glutamine, and GLUL inhibition can transfer M2-like TAMs into M1-like phenotype by increasing glycolytic flux and succinate availability (51).

### Regulated by Immune Cells

TAMs can be regulated by other immune cells, such as Treg cells, MDSCs and B cells. IFN-γ is the main cytokines responsible for inhibiting M2-like TAM. Treg cells can inhibit IFN-γ secreted by CD8^+^ T cells, which will prevent the activation of fatty acid synthesis that mediated by sterol regulatory element binding protein 1 (SREBP1) in immunosuppressive M2-like TAM. Therefore, Treg cells indirectly but selectively maintain M2-like TAM metabolic adaptability, mitochondrial integrity and survival rate (56). In addition, MDSCs also regulate TAM differentiation and promote tumor proliferation by downregulation of STAT3 (57). Besides, B cells are the key factors determining the tumor promoting function of TAMs. B cells can induce M2b macrophage polarization in human HCC (58), as well as suppress other immune cells, such as CD8+ T cells and M1 macrophages in the tumor microenvironment and promote the proliferation of cancer cell (59). Depletion of B cells prevented generation of M2b, increased the activity of anti-tumor T cell response, and reduced tumor growth.

### Regulation by Other Factors

There are also some other factors of tumor microenvironment that can regulate TAMs function. Autophagy in the tumor microenvironment can provide essential nutrients, nucleotides, and amino acids to the tumor cells, facilitating tumor growth (60). Autophagy proteins in myeloid cells in the tumor microenvironment help to activate TAM by influencing LAP and mediate immunosuppression of T lymphocytes (61). In non-alcoholic fatty liver disease (NAFLD), NLRC4 contributes to the polarization of TAM to M2 type and the production of IL-1β and VEGF, thereby promoting the growth of tumor (62). Moreover, C-Maf transcription factor is the main regulator of cancer-promoting TAM polarization. C-Maf can promote the immunosuppressive activity of TAMs and control its metabolic process (63).

## Targeting TAMs for Cancer Treatment

TAMs are one of the most important components of the tumor immunosuppression microenvironment with high degree of plasticity. TAMs have both M1 and M2 type and have the potential ability of repolarization to M1 type macrophages. Therefore, targeting TAMs is a new cancer treatment strategy, including limiting monocytes recruitment, targeting TAMs activation, reprogramming TAMs into anti-tumor activity, and targeting TAMs specific markers (**Figure 2**).

### Limiting Monocyte Recruitment

One of the strategies for targeting TAMs is to block monocyte recruit to tumor tissue. Tumor cells recruit CCR2-expressing monocytes from the peripheral blood to the tumor site by releasing CCL2 and these recruit CCR2-expressing monocytes will finally mature into TAMs, which accelerate the tumor progress. Thus, targeting CCL2-CCR2 axis is a very effective method of cancer therapy. Blocking the CCL2-CCR2 axis could greatly reduce the incidence of tumors by preventing TAMs recruitment and enhance the anti-tumor efficacy of CD8+ T cells in the tumor microenvironment (64).

CSF1 signaling pathway plays a key role in the production of bone marrow monocytes and the polarization of TAMs in tumor tissues. CSF1 produced by tumor cells caused down-regulation of granulocyte-specific chemokine expression in HDAC2-mediated cancer-associated fibroblasts (CAF), thereby limiting the migration of monocytes to tumors. The combination of CSF1R inhibitor and CXCR2 antagonist can prevent granulocytes from infiltrating the tumor, showing a strong anti-tumor effect (65). Also, combination of anti-PD-1 and anti-CSF1R antibodies induces melanoma regression in mice (66).

### Targeting TAM Activation

Targeted activation of TAMs is an effective tumor treatment method. One of them is inhibiting TAMs from promoting tumor cell activation. Epidermal cell growth factor (EGF) secreted by TAM activates EGFR on tumor cells, which in turn upregulates VEGF (vascular endothelial growth factor)/VEGFR signaling in surrounding tumor cells, thereby promoting the proliferation and migration of tumor cells. EGFR blockade or ICAM-1 (intercellular adhesion molecule) antibody neutralization in TAM reduced the occurrence of ovarian cancer in mice (25).

Another effective tumor treatment method is blocking inhibitory receptor signals on TAMs that promote phagocytosis and antigen presentation function. Tumor cells highly express CD47, which restricts the ability of macrophages to engulf tumor cells through the signal regulatory protein alpha (SIRPα) -CD47 signal. The destruction of the SIRPα-CD47 signal axis is effective against various brain tumors including glioblastoma multiforme (GBM) by inducing tumor phagocytosis (67). Leukocyte immunoglobulin-like receptor subfamily B (LILRB) family is a class of inhibitory receptors expressed by myeloid cells, and its ligands are MHCI-like molecules (68). LILRB1 is up-regulated on the surface of TAM, and the MHCI-like component β2-microglobulin expressed by cancer cells can directly protect it from being engulfed. Therefore, blocking MHC I molecules or LILRB1 can enhance TAM phagocytosis (69).

Targeting pre-tumor myeloid cells at the metabolic level is another therapeutic strategy. Immunosuppressive phenotype of TAMs is controlled by long-chain fatty acid metabolism (especially unsaturated fatty acids), which makes BMDMs polarized into M2 phenotypes with strong inhibitory ability. Therefore, chemical inhibitors can effectively block TAM polarization *in vitro* and tumor growth *in vivo* (70).

### Reprogramming TAMs Into Anti-Tumor Activity

One of the key characteristics of macrophages is their plasticity, which allows them to change the phenotype according to the tumor microenvironment. Therefore, reprogramming TAMs into an anti-tumor phenotype is a very promising tumor treatment strategy. Anti-tumor macrophages (M1 type) have abilities to clear and destroy tumor cells. RP-182 can selectively induce conformational switching of the mannose receptor CD206 expressed on TAM expressing the M2 phenotype, reprogramming M2-like TAM into anti-tumor M1-like TAM phenotype (71). Another finding shows that serine/threonine protein kinase 1 (RIP1) interacting with receptors in TAMs in pancreatic ductal adenocarcinoma (PDA) is up-regulated. Targeting RIP1, which act as a checkpoint kinase, reprogram TAM toward MHCII^hi^ TNFα^+^ IFNγ^+^ phenotype (72).

### Targeting Immune Inhibitory Molecules on TAMs

Targeting immune inhibitory molecules on TAMs is also an effective method. Blocking of MerTK leads to the accumulation of apoptotic cells in tumor cells and triggers a type I interferon response. MerTK blockade increases tumor immunogenicity and enhances anti-tumor immunity. Treatment of tumor-bearing mice with anti-MerTK antibodies can stimulate T cell activation and synergize with anti-PD-1 or anti-PD-L1 therapy (73). PD-1-PD-L1 therapy can also work by direct action on macrophages. Both mouse and human TAM express PD-1. The expression of TAM PD-1 is negatively correlated with the phagocytic ability against tumor cells, and blocking PD-1-PD-L1 *in vivo* will increase the phagocytosis of macrophages, reduce tumor growth, and rely on macrophage-dependent ways to prolong the survival of mice in cancer models (74).

## Concluding Remarks

Under the effect of the tumor microenvironment, TAMs are tamed by tumor cells and has become a promoter of tumor growth. Studies have shown that TAMs have a significant role in promoting the development and progress of tumors. Therefore, how to inhibit the tumor-promoting roles of TAMs will provide new clues for future tumor therapy. However, a number of key questions remain to be answered, including mechanisms of TAM development, key factors that drive phenotypic changes of TAMs in the tumor microenvironment. Recent pre-clinical and clinical studies aiming at targeting TAMs for cancer treatment have shown inspiring results. TAM-targeting therapy represents a promising treatment of cancer patients in the future.

## Author Contributions

YP and YY analyzed the data and wrote the paper. XW and TZ edited the manuscripts. All authors contributed to the article and approved the submitted version.

## Conflict of Interest

The authors declare that the research was conducted in the absence of any commercial or financial relationships that could be construed as a potential conflict of interest.
