# Corrigendum: Tumor-Associated Macrophages in Tumor Immunity

**DOI:** 10.3389/fimmu.2021.775758

**Published:** 2021-12-10

**Authors:** Yueyun Pan, Yinda Yu, Xiaojian Wang, Ting Zhang

**Affiliations:** ^1^ Department of Radiation Oncology, Second Affiliated Hospital, School of Medicine, Zhejiang University, Hangzhou, Zhejiang, China; ^2^ Institute of Immunology, School of Medicine, Zhejiang University, Hangzhou, Zhejiang, China

**Keywords:** tumor-associated macrophages, regulation, immunosuppression, tumor microenvironment, tumor therapy

The corresponding authors were incorrectly listed in the published work. In addition to **Ting Zhang**, author **Xiaojian Wang** is also a corresponding author.

The authors apologize for this error and state that this does not change the scientific conclusions of the article in any way. The original article has been updated.

## Publisher’s Note

All claims expressed in this article are solely those of the authors and do not necessarily represent those of their affiliated organizations, or those of the publisher, the editors and the reviewers. Any product that may be evaluated in this article, or claim that may be made by its manufacturer, is not guaranteed or endorsed by the publisher.

